# A decade of atrial fibrillation ablation: Shifts in patient characteristics and procedural outcomes

**DOI:** 10.1007/s12471-017-1019-7

**Published:** 2017-07-17

**Authors:** C. Teunissen, N. Clappers, R. J. Hassink, J. F. van der Heijden, F. H. Wittkampf, P. Loh

**Affiliations:** Division of Heart and Lungs, Department of Cardiology, University Medical Center Utrecht, Utrecht, The Netherlands

**Keywords:** Radiofrequency catheter ablation, Pulmonary vein antrum isolation, Atrial fibrillation, Outcomes

## Abstract

**Background:**

Over the past decade, radiofrequency catheter ablation (RFCA) of atrial fibrillation (AF) has evolved into a frequently performed procedure. The aim of this study was to monitor changes in patient characteristics, procedural characteristics, outcomes and complications over the past 10 years.

**Methods:**

All consecutive patients who underwent primary RFCA treatment of AF in the University Medical Center Utrecht from 2005–2015 were included. In all patients, the primary ablation strategy was pulmonary vein (PV) antrum isolation without additional substrate modification. Baseline patient and procedure characteristics, and 1‑year follow-up data of 975 patients were prospectively collected.

**Results:**

In 2005, 73.4% of patients suffered from paroxysmal AF, which decreased to 45.3% in 2014. Mean age increased from 54 ± 9 to 61 ± 10 years and CHA_2_DS_2_-VASc score ≥2 from 18 to 40.6%. History of AF decreased significantly from 7 to 4 years. Mean procedure duration was 237 ± 53 min in 2005 and 163 ± 41 min in 2014. Fluoroscopy time significantly decreased from 41 ± 17 to 19 ± 8 min and total radiation exposure from 465 (263–687) to 210 (118–376) mGy. One-year success remained similar (2005: 55.6%, 2014: 54.8%), as did the amount of PV reconnection observed during redo procedures. Due to a marked reduction in vascular complications and moderate PV stenosis, the total complication rate decreased significantly.

**Conclusion:**

Over the past decade, AF ablation has increasingly been performed in older patients with persistent AF and more comorbidity. Moreover, it has been performed earlier after AF diagnosis. Although several performance parameters, such as procedure duration and complication rate, improved, 1‑year single procedure success remained unchanged.

## Introduction

Radiofrequency catheter ablation (RFCA) is a relatively novel treatment option for atrial fibrillation (AF). Less than two decades ago, RFCA treatment was limited to either experimental ablations in both atria or palliative ablation of the atrioventricular node [1, 2]. However, by identification of the pulmonary veins (PVs) as the most important trigger of AF, a potential curative target for RFCA arose [3]. Nowadays, the cornerstone of RFCA treatment is antrum isolation (PVAI) [4].

Over the past decade, RFCA treatment has matured. It has evolved from a fairly unfamiliar treatment to a frequently performed, widely accepted procedure. For patients with symptomatic paroxysmal AF refractory or intolerant to at least one Class 1 or 3 antiarrhythmic drug, RFCA advanced to a Class 1 recommended treatment [4]. Nevertheless, RFCA outcomes remain imperfect. Single procedure success is modest and redo procedures are often needed [5, 6].

Technical developments, increased physician experience and scientific findings may have led to shifts in patient selection and, as a consequence, patient characteristics may have changed over time. Furthermore, these developments may have affected procedural outcomes. Yet, whether promising technical developments and results from clinical trials truly translate into clinical benefit remains uncertain. Therefore, monitoring of changes in real-world procedural outcomes may be meaningful. Moreover, it guides physicians in properly informing patients on the expected outcomes of RFCA.

The goals of this single-centre study were to [1] analyse shifts in patient characteristics, procedural characteristics, outcomes and complications over the past decade and [2] describe procedural and technical developments that may have led to these shifts.

## Methods

### Study population

All consecutive patients with symptomatic, drug-refractory or drug-intolerant AF who underwent first PVAI in the University Medical Center Utrecht from 2005 to 2015 were included in this analysis. Baseline patient and procedure characteristics, and 1‑year follow-up data were prospectively collected. This study was approved by the institutional review board.

### Baseline characteristics

Prior to the first ablation, baseline characteristics were collected and included: sex, age, type of AF, history of AF (years since AF was diagnosed), risk factors for cardiovascular disease, CHA_2_DS_2_-VASc score, congestive heart failure, structural heart disease and left atrial (LA) size (end-systolic LA diameter in the parasternal long axis view on echocardiography). AF type was classified as paroxysmal, persistent or longstanding persistent according to the HRS/EHRA/ECAS 2012 Consensus Statement on Catheter and Surgical Ablation of AF [4].

### Electrophysiological study and ablation strategy

Pre-ablation workup, electrophysiological study and post-ablation care have been described in detail elsewhere [6, 7]. In short, a three-dimensional cardiac mapping system (EnSite NavX and Velocity; St. Jude Medical Inc. or Carto; Biosense Webster) was used to obtain a three-dimensional reconstruction of the left atrium, including the left atrial appendage and the PVs. The left and right PVs were widely encircled at their antrum using an irrigated tip catheter (ThermoCool irrigated tip catheter, Biosense Webster, ThermoCool SmartTouch, Biosense Webster, and TactiCath; St. Jude Medical). During the complete duration of the study, primary ablation strategy was PVAI without additional ablation of complex fractionated atrial electrograms and without linear ablation lesions in the left atrium.

**Fig. 1:**
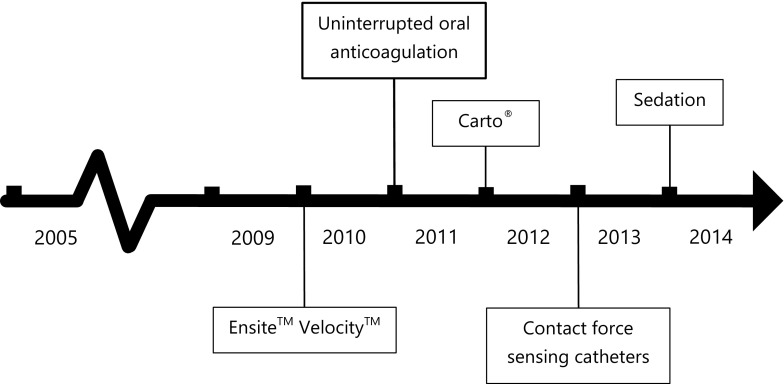
Timeline of changes in procedural care In 2010, EnSite NavX has been upgraded to EnSite Velocitiy. From 2011 onwards, oral anticoagulation therapy was uninterrupted throughout the procedure. Since 2012, CARTO, Biosense Webster has been applied besides EnSite (St. Jude Medica)l as a three-dimensional mapping system. The use of adenosine has gradually been introduced since 2012 and was left to the physician’s discretion. Contact force catheters (SmartTouch (Biosense Webster) or TactiCath (St. Jude Medical)) have been used since 2013. Until 2013, electrophysiological study was performed in a non-sedated state. After 2013, electrophysiological study was performed under procedural sedation with propofol and remifentanil

During this study, some major changes in procedural care have been implemented. An overview is shown in Fig. 1.

### Follow-up of patient

Patients were seen at the outpatient clinic at 3, 6 and 12 months after the procedure. The patient’s rhythm status was evaluated using patient history and a 12-lead electrocardiogram at every visit and with additional 48-hour Holter recordings at 3 and 6 months. After 3 to 6 months of follow-up, magnetic resonance imaging or computed tomography of the heart was routinely performed to rule out PV stenosis.

### Outcomes

One-year success was defined according to the 2012 HRS/EHRA/ECAS consensus statement as complete freedom from AF, atrial flutter (AFl) or atrial tachycardia recurrences following the 3‑month blanking period in the absence of Class I and III antiarrhythmic drug therapy [4].

### Statistics

Patient characteristics were reported as percentages, counts, median (25th to 75th percentile) or mean ± SD, as appropriate. For categorical variables, significant variation over time was determined using the chi-square test. For continuous variables, significant variation over time was analysed using ANOVA in case of homogeneous variance or Kruskal-Wallis *H*-test if not homogeneous. The CHA_2_DS_2_VASc score was dichotomised at the clinically relevant cut-off value of ≥2.

In order to identify significant predictors of recurrence of atrial tachyarrhythmias after PVAI, multivariate logistic regression analysis was performed. Predictors for the multivariate prediction model were selected based on known or expected clinical relevance and comprised all baseline characteristics and year of ablation. A *p*-value <0.05 was considered statistically significant. Statistical analyses were performed using SPSS 21.0 (IBM, Armonk, NY, USA).

## Results

From 2005 to 2015, 975 consecutive patients underwent primary PVAI. The number of patients undergoing RFCA gradually increased over the years (Tab. 1).

**Table 1:** Patient characteristics (*n* = 975)

	2005 *n* = 64	2006 *n* = 51	2007 *n* = 96	2008 *n* = 89	2009 *n* = 98	2010 *n* = 105	2011 *n* = 118	2012 *n* = 115	2013 *n* = 133	2014 *n* = 106	*p*-value
Male sex	79.9	74.5	76.0	80.9	72.4	74.3	74.6	75.7	76.6	72.4	0.951
Age, y	54 ± 9	55 ± 9	57 ± 9	58 ± 10	58 ± 10	57 ± 11	59 ± 10	60 ± 10	58 ± 10	61 ± 10	**0.001**
*AF type*											**<0.001**
Paroxysmal	73.4	62.7	70.8	43.8	56.1	64.8	58.8	54.8	60.2	45.3	**–**
Persistent	21.9	25.5	17.7	37.1	30.6	21	29.7	39.1	35.3	52.8	**–**
LS persistent	4.7	11.8	11.5	19.1	13.3	14.3	11.9	6.1	4.5	1.9	**–**
History of AF, y	7 (4–12)	5 (2–9)	5 (3–8)	5(2–11)	5 (3–9)	5 (2–10)	5 (2–10)	3 (1–7)	4 (2–9)	4 (2–8)	**<0.001**
BMI, kg/m^2^	26 (23–28)	26 (23–29)	26 (25–29)	26 (25–29)	26 (24–30)	26 (24–29)	26 (24–29)	27 (25–30)	26 (24–30)	27 (24–30)	0.599
CHA_2_DS_2_-VASc ≥2	18.0	24.5	32.3	33.7	32.3	36.9	37.3	46.1	32.3	40.6	**0.023**
Hypertension	31.3	19.6	30.2	32.6	36.7	37.1	33.9	42.6	43.6	40.6	0.077
DM	0	9.8	4.2	7.9	6.1	5.7	5.9	13.9	6.0	10.4	**0.043**
Atrial flutter	28.1	23.5	35.4	31.5	25.5	25.7	25.4	31.3	21.8	30.2	0.476
CHF	1.6	0	1.0	6.7	9.2	7.6	6.8	2.6	2.3	3.8	**0.024**
Lone AF	26.6	23.5	21.9	24.7	18.4	24.8	28.0	23.5	18.0	13.2	0.193
SHD	6.3	11.8	13.2	20.3	12.4	12.4	17.8	17.4	12.0	18.9	0.545
LA size, mm	42 ± 6	43 ± 6	44 ± 6	44 ± 7	43 ± 6	43 ± 6	42 ± 7	42 ± 8	43 ± 8	42 ± 7	0.257

Data are expressed as percentages, mean+SD or median (25th–75th percentile)

*AF* atrial fibrillation, *LS* persistent longstanding persistent, *BMI* body mass index, *DM* diabetes mellitus, *AFl* atrial flutter, *CHF* congestive heart failure, *SHD* structural heart disease, *LA* left atrial

### Patient characteristics

**Fig. 2:**
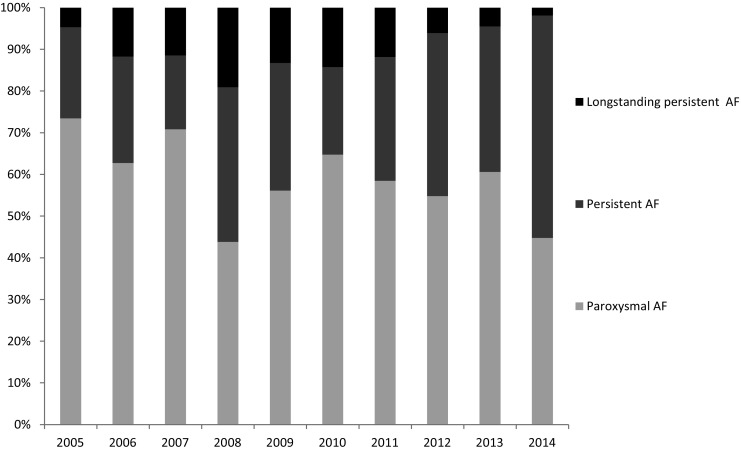
Distribution of atrial fibrillation types over the past decade

Dispersion of patient characteristics over the past decade is shown in Tab. 1. Mean age at ablation increased from 54 ± 9 years in 2005 to 61 ± 10 years in 2014 (*p* = 0.001). In 2005, 73.4% of patients suffered from paroxysmal AF, which decreased to 45.3% in 2014 (*p* < 0.001). Incidence of long-standing persistent AF resembled a parabolic form (Fig. 2). History of AF (years since diagnosis) significantly decreased from 7 to 4 years. In 2005 18% of patients had a CHA_2_DS_2_-VASc score ≥2, in 2014 40.6% (*p* = 0.023).

### Procedural characteristics and outcomes

**Fig. 3:**
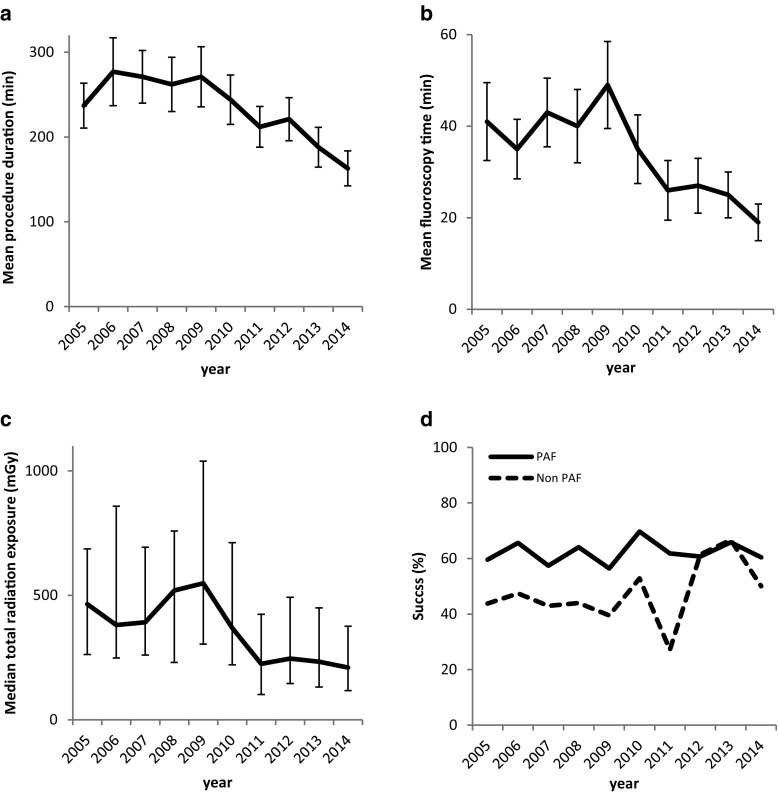
Procedural characteristics and outcomes of primary pulmonary vein antrum isolation over the past decade. **a** Mean procedure duration in minutes, **b** Mean fluoroscopy time in minutes, **c** Median total radiation exposure in mGy, **d** Single procedure success (complete freedom from atrial tachyarrhythmias recurrences off anti arrhythmic drugs) after one year of follow-up in paroxysmal atrial fibrillation and non-paroxysmal atrial fibrillation

Tab. 2 shows procedural characteristics and outcomes of the past 10 years. In 2005, mean procedure duration from femoral vein access to catheter withdrawal was 237 ± 53 min, which decreased to 163 ± 41 min in 2014. The main decrease in procedure duration was noticed in 2013 after introduction of contact force (CF) sensing catheters (Fig. 3). Fluoroscopy time significantly decreased from 41 ± 17 to 19 ± 8 min and total radiation exposure from 465 (263–687) to 210 (118–376) mGy (Fig. 3). A consistent decrease in fluoroscopy time and radiation exposure was observed from 2010 onwards, after implementation of novel three-dimensional navigation systems and later CF catheters (Fig. 3).

**Table 2:** Procedural characteristics and outcomes (*n* = 975)

	2005 *n* = 64	2006 *n* = 51	2007 *n* = 96	2008 *n* = 89	2009 *n* = 98	2010 *n* = 105	2011 *n* = 118	2012 *n* = 115	2013 *n* = 133	2014 *n* = 106	*p*-value
Procedure duration, min	237 ± 53	277 ± 80	271 ± 62	262 ± 64	271 ± 71	244 ± 58	212 ± 48	221 ± 51	188 ± 47	163 ± 41	**<0.001**
Fluoroscopy time, min	41 ± 17	35 ± 13	43 ± 15	40 ± 16	49 ± 19	35 ± 15	26 ± 13	27 ± 12	25 ± 10	19 ± 8	**<0.001**
Total radiation exposure, mGy	465 (263–687)	381 (249–858)	392 (260–694)	520 (231–759)	549 (304–1039)	369 (221–712)	225 (102–424)	246 (146–492)	234 (132–450)	210 (118–376)	**<0.001**
1 year success											
PAF	59.6	65.6	57.4	64.1	56.4	69.7	61.8	60.7	65.8	60.4	0.916
Non-PAF	43.8	47.4	42.9	44.0	39.5	52.8	27.1	61.5	66.7	50.0	**0.010**
Redo <1 year	7.8	13.7	14.6	18.0	11.2	15.2	23.7	14.8	16.5	18.9	0.240
Redo <2 years	28.6	20.4	35.8	36.0	29.6	23.8	35.3	27.2	23.3	37.7	0.091
PV reconnection	94.4	80.0	100	87.5	96.6	84.0	85.4	90.3	93.5	87.2	0.357

Data are expressed as percentages, mean + SD or median (25th–75th percentile). Procedure duration was calculated from femoral vein access to catheter withdrawal. Success was defined as complete freedom from atrial fibrillation, atrial flutter or atrial tachycardia recurrences. Redo procedures and pulmonary vein reconnection are analysed per year of the index procedure

*PAF* paroxysmal atrial fibrillation, *PV* pulmonary vein, *mGy* milligray

Of the 975 included patients, 19 (1.9%) were lost to follow-up. Of the remaining 956 patients, 55.3% were completely free of atrial tachyarrhythmia recurrence off antiarrhythmic drug therapy 1 year after primary PVAI. During the entire study duration, most recurrences were based on AF. Only in 4.7% of patients recurrences we based on left-sided atrial tachycardia or AFl. This was consistent over the years.

Over the past decade, the overall success rate did not change. In 2005, one-year success was 55.6%, in 2014 this was 54.8%. Also in patients with paroxysmal AF, success remained similar. In patients with non-paroxysmal AF, 1‑year success was lower and demonstrated a more fluctuating course (Fig. 2). Independent predictors of recurrences of atrial tachyarrhythmia after primary PVAI were female sex (HR: 1.58, 95% CI: 1.08–2.31, *p* = 0.018), persistent AF (HR: 1.50, 95% CI: 1.09–2.07, *p* = 0.013), longstanding persistent AF (HR: 2.23, 95% CI: 1.37–3.62, *p* = 0.001) and history of AF (HR: 1.04, 95% CI: 1.01–1.06, *p* = 0.008). Year of ablation was no significant predictor for recurrence (HR: 0.96, 95% CI: 0.91–1.02, *p* = 0.190). So, independent of patient characteristics, procedure success did not change over a 10-year period.

The number of redo procedures within 2 years after the primary ablation procedure and the number of patients with reconnection of at least one PV observed during these redo procedures (analysed per date of the index procedure) remained unchanged over the past decade (Tab. 2).

### Complications

The overall complication rate has significantly decreased in the past decade (Tab. 3). Vascular complications, as well as moderate PV stenosis (50–70% diameter reduction), significantly decreased. Severe PV stenosis (>70% diameter reduction) occurred in 6 patients, all in 2011 and 2012. Of all the patients with a moderate or severe PV stenosis, one was symptomatic. This patient suffered from a total occlusion of the left inferior PV and a severe stenosis of the left superior PV. Both PVs were successfully stented. Cerebral vascular accident occurred once in 2006 and a suspected transient ischaemic attack twice in 2007. After 2007, no thromboembolic complications occurred (*p* = 0.049). Incidence of other complications (e. g. tamponade) did not change over time. In the 10 years, one patient died suddenly 5 weeks after PVAI due to cardiac tamponade caused by a peri-epicarditis. No atrial-oesophageal fistula occurred.

**Table 3:** Complications (*n* = 975)

	2005 *n* = 64	2006 *n* = 51	2007 *n* = 96	2008 *n* = 89	2009 *n* = 98	2010 *n* = 105	2011 *n* = 118	2012 *n* = 115	2013 *n* = 133	2014 *n* = 106	*p*-value
Total	25.0	13.7	9.4	11.2	7.1	6.7	11.0	7.0	1.5	8.5	**<0.001**
Vascular	7.8	7.8	5.2	9.0	1.0	1.9	0.8	1.7	0	2.8	**0.001**
Tamponade	0	0	1.0	0	3.1	1.9	0.8	0	0	1.9	0.255
CVA/TIA	0	2.0	2.1	0	0	0	0	0	0	0	**0.049**
PV stenosis											
Moderate	15.6	3.9	1.0	2.2	3.1	2.9	5.9	3.4	0.8	2.9	**<0.001**
Severe	0	0	0	0	0	0	3.4	1.7	0	0	**0.008**
Mortality	0	0	0	0	0	1.0	0	0	0	0	0.507
Other	1.6	0	0	0	0	0	0	0	0.8	0.9	0.574

Data are expressed as percentages. Moderate PV stenosis: 50–70% diameter reduction, Severe PV stenosis: >70% diameter reduction. Other includes: air embolus in a coronary artery (*n* = 1 in 2005), phrenic nerve paresis (*n* = 2)

*CVA* cerebral vascular accident, *TIA* transient ischaemic attack, *PV* pulmonary vein

## Discussion

### Main outcomes

In this study, we have highlighted real-world shifts in patient characteristics and procedural outcomes over the past decade. As the use of RFCA increased and it gained the referring physician’s trust, it was employed in a more heterogeneous patient population and earlier after patients were diagnosed with AF. It is increasingly being performed in older patients with more comorbidity and persistent AF. Procedure duration, fluoroscopy time and radiation dose substantially have decreased, likely due to implementation of novel three-dimensional cardiac mapping systems and CF sensing catheters. Due to a decrease in vascular complications and moderate PV stenosis, the total complication rate has significantly decreased. Remarkably, 1‑year single procedure success and the rate of PV reconnection observed during redo procedures have remained similar.

### Shifts in atrial fibrillation types

Ablations have increasingly been performed in patients suffering from persistent AF, while there was a decrease in patients with long-standing persistent AF. In the past decade, disappointing results of RFCA in long-standing persistent AF have been presented in several studies, despite various ablation strategies [6, 8, 9]. These results may have led to a more reluctant approach in this subset of patients. On the other hand, possibly less patients reached 1 year of continuous AF. In more recent years, efforts were made to treat patients with a few months of continuously present AF with priority.

### Complications

Although the incidence of tamponade and thromboembolic complications were comparable with previous studies, the rate of PV stenosis was higher in our study [10]. In contrast to our approach, other institutions only screened for PV stenosis in case of symptoms. True incidence of PV stenosis is therefore likely underestimated in literature.

In the early years of this study, oral anticoagulation therapy was interrupted preceding the ablation procedure and patients were bridged with subcutaneous low-molecular-weight heparin. Several studies, however, revealed that uninterrupted oral anticoagulation therapy throughout the procedure resulted in less bleeding and thromboembolic complications [11]. We have adopted this strategy, which may have resulted in the decrease of vascular complications.

### Success of catheter ablation treatment

Regarding the unchanged success rate, two observations stand out: the rate of PV reconnection encountered during redo procedures has not decreased and success rates are lower in patients with non-paroxysmal-AF. To increase the efficacy of the contemporary procedure, two major challenges have to be overcome: 1) achieving durable PV isolation by creating permanent, transmural ablation lesions and 2) sufficient understanding, identification and ablation of the atrial substrate in a subset of patients with non-paroxysmal AF.

### Durable isolation of the pulmonary veins

Prevention of PV reconnection by creating durable ablation lesions has been extensively investigated and several promising technical and scientific breakthroughs have emerged. First, CF sensing catheters have been developed. In RFCA, insufficient CF may result in ineffective ablation lesions, whereas excessive CF may result in complications such as cardiac tamponade. Despite the fact that safety and applicability of CF catheters has been demonstrated [12], two randomised studies failed to confirm superiority of CF catheters over non-CF catheters [13, 14]**. **However, both studies indicated in subgroup analysis that sufficient CF resulted in favourable outcomes [13, 14]. Furthermore, two studies showed that procedure duration, fluoroscopy time and radiation dose were significantly reduced by the use of CF catheters.[13, 15]. Besides CF sensing catheters, advanced three-dimensional navigation systems allowing more anatomical details have shown to significantly reduce fluoroscopy time [16].

The use of adenosine formed another encouraging innovation. In the ADVICE trial, adenosine-guided PV isolation led to a significant absolute risk reduction of 27.1% in arrhythmia recurrence in patients suffering from paroxysmal AF [17].

Last, other ablation techniques might increase persistence of ablation lesions. Although robotic-assisted RFCA, second generation cryoballoon and laser balloon ablations form appropriate alternatives for manual RFCA, to date, none of these methods have proven to be superior [18–22].

### Significance of the study findings

We have revealed that success of the RFCA did not improve significantly despite growing experience and technical developments. For over a decade, our procedural endpoint has been persistent isolation of the PVs after a 30-minute observation period. However, this is not an adequate predictor of permanent isolation. Future research should focus on techniques that will allow transmural and permanent ablation lesions. Furthermore, understanding of the changing patient characteristics may facilitate designs of future studies.

### Limitations

This study has some limitations. It is a single-centre cohort study. Our results may not be translated to other institutions. Nevertheless, procedural outcomes may be representative for institutions with a similar approach.

As we did not routinely perform 48-hour Holter recordings after 12 months of follow-up, some asymptomatic AF episodes may have been missed.

Changes in procedure characteristics and outcomes are multifactorial. Since our study was a descriptive study, it was not designed to study the effect of all separate contributing factors. Besides technical developments, other factors such as personal learning curves can attribute. However, it is challenging to estimate the exact influence of personal learning curves. Over the past, decade ablations were performed by starting and experienced electrophysiologists as well as fellows in our tertiary centre.

## Conclusion

Over the past decade, RFCA of AF has become less reserved for young patients with paroxysmal AF and more often performed in patients with more comorbidity and persistent AF. Furthermore, RFCA was performed earlier after patients were diagnosed with AF. Although several ablation performance parameters such complication rate, procedure duration and radiation dose have improved, one-year single procedure success remained unchanged.
